# *Arabidopsis thaliana *outer ovule integument morphogenesis: Ectopic expression of *KNAT1 *reveals a compensation mechanism

**DOI:** 10.1186/1471-2229-8-35

**Published:** 2008-04-14

**Authors:** Elisabeth Truernit, Jim Haseloff

**Affiliations:** 1 University of Cambridge, Department of Plant Sciences, Downing Site, Cambridge CB2 3EA, UK; 2 INRA, Centre de Versailles, Institut Jean-Pierre Bourgin, Laboratoire de Biologie Cellulaire, Route de St-Cyr, 78026 Versailles cedex, France

## Abstract

**Background:**

The *Arabidopsis *outer ovule integument is a simple two-cell layered structure that grows around the developing embryo and develops into the outer layer of the seed coat. As one of the functions of the seed coat is the protection of the plant embryo, the outer ovule integument is an example for a plant organ whose morphogenesis has to be precisely regulated.

**Results:**

To better characterise outer ovule integument morphogenesis, we have isolated some marker lines that show *GFP *expression in this organ. We have used those lines to identify distinct cell types in the outer integument and to demonstrate similarities between leaves and the outer integument. Using confocal microscopy, we showed that cell sizes and shapes differ between the two cell layers of the outer integument. Expression of *KNAT1 *in the integuments leads to extra cell divisions specifically in the outer layer of the outer integument. This is being compensated for by a decrease of cell volume in this layer, thus showing that mechanisms exist to control proper ovule integument morphogenesis.

**Conclusion:**

The *Arabidopsis *outer ovule integument can be used as a good model system to study the basic principles of plant organ morphogenesis. This work provides new insights into its development and opens new possibilities for the identification of factors involved in the regulation of cell division and elongation during plant organ growth.

## Background

**Figure 1:**
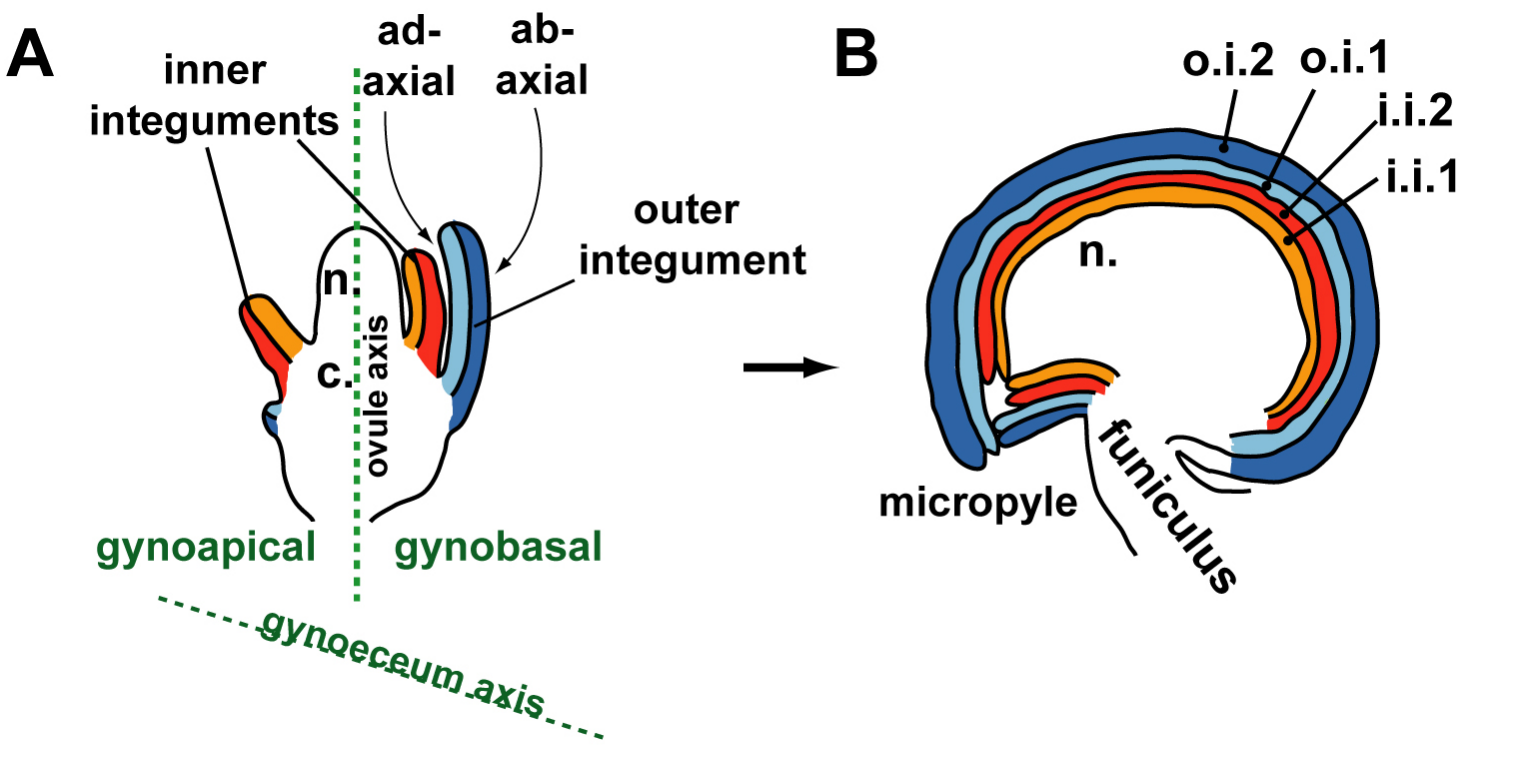
**The development of ovule integuments in *Arabidopsis***. **(A) **Two inner and one outer integument grow out from the chalaza (c) during early ovule development. **(B) **Ovule at stage of fertilization: Integuments have grown around nucellus (n), i.i.1: inner (adaxial) layer of inner integument, i.i.2: outer (abaxial) layer of inner integument, o.i.1: inner (adaxial) layer of outer integument, o.i.2: outer (abaxial) layer of outer integument.

Fertilised ovules develop into seeds that contain the plant embryo. In *Arabidopsis thaliana*, three distinct regions can be identified along the proximal-distal axis of the ovule primordium (Figure 1). The most proximal structure of the primordium is the funiculus, which connects the primordium to the placenta. At the distal end of the primordium lies the nucellus in which the megaspore mother cell develops. The chalaza in the central zone of the primordium initiates two integuments, each composed of two cell layers [1,2]. During ovule development, the two integuments grow around the nucellus and, after fertilization, develop into the seed coat that encloses the embryo (Figure 1). Whereas the inner integument initially develops as a radially symmetrical structure that surrounds the nucellus, the outer integument grows only from the side of the ovule primordium that faces the basal end of the carpel (gynobasal side) [1,2]. The outer integument remains two-cell layered throughout seed development [1,2]. At later stages of seed development, cells of the abaxial (outer) layer of the outer integument differentiate terminally into highly specialized seed coat cells that contain polysaccharide mucilage [3,4].

The integuments are the only lateral organs produced by the ovule. The evolutionary origin of the integuments is still a matter of debate. The telome theory suggests that integuments originated from the fusion of sterile or fertile branches (telomes) [5,6]. It is generally believed that the inner and outer integument derived independently. While the inner integument most likely originated directly from the fusion of telomes or sporangiophores, the outer integument is believed to have developed later from a cupule, a leaf-like structure surrounding one or more ovules [7,8].

The development of the *Arabidopsis *outer ovule integument involves the same basic processes required for the formation of other determinate lateral plant organs, such as leaves. The outer ovule integument is an example for an organ of determinate growth and characteristic form in which the rate and direction of cell division and elongation needs to be precisely regulated. Asymmetric growth and differentiation are also essential features of its development. In case of the integuments, proper morphogenesis is especially critical, as an improper curvature or closure would lead to seeds with embryos that are not sufficiently protected. However, it seems that integument extension is relatively sensitive to alterations in cell division or cell expansion. Mutations in *SHORT INTEGUMENTS2 *(*SIN2*), for example, lead to shorter integuments due to a reduction of cell number [9]. The result of mutations in *SIN1/DCL1*, on the other hand, show reduced integument size due to a lack of cell expansion [1,10,11].

Because of its simple two-layered structure, the outer integument is an ideal organ for the study of the basic principles of plant morphogenesis. For this a better characterisation of outer integument growth and cell fates within the integument is required. To address this, we have identified *Arabidopsis *enhancer-trap lines with specific expression of the gene for green fluorescent protein (GFP) in distinct domains of the outer integument. These lines provided good markers for the characterisation of cell proliferation and differentiation during development of the outer integument. KNAT1 is a homeodomain protein that is normally expressed in the shoot apical meristem (SAM), and which alters leaf morphology when ectopically expressed in leaves [12,13]. Misexpression of *KNAT1 *caused increased cell division specifically in the abaxial layer of the outer integument and showed that compensatory mechanisms exist in the outer integument to ensure its proper morphogenesis.

## Results

### A screen for marker lines for the study of outer integument development

To obtain markers for the study of outer ovule integument development, a population of 400 *Arabidopsis *C24 enhancer-trap lines [14] was screened for *GFP *expression in the outer ovule integument. Six lines showed stable patterns of *GFP *expression in this tissue. In seed coats of seeds that contained walking stick stage embryos, *GFP *expression in three of the lines (KS059, KS110, KS151) was seen throughout the outer layer of the outer integument, one line (KS149) showed expression in both outer integument cell layers, and two lines (M214, M237) showed *GFP *expression that was restricted to the micropylar end of the outer layer of the outer integument. Four of the lines (KS110, KS151, KS149, M237) were chosen for a more detailed analysis of *GFP *expression patterns throughout ovule and seed development using confocal laser-scanning microscopy (ovules and seeds of three independent plants, n ≥ 5/stage).

To describe *GFP *expression patterns in those four lines we will follow a recent suggestion by Skinner et al. [15]. We will use the term "gynobasal" to refer to the side of the ovule primordium that faces the base (receptacle) of the carpel, and the term "gynoapical" for the side that faces the apical (stigma) side of the carpel. The terms "abaxial" and "adaxial" will be used to refer to the polarity of the lateral organs of the ovule, i.e. the integuments (see Figure 1).

### Markers for the adaxial-abaxial polarity in the outer integument

**Figure 2:**
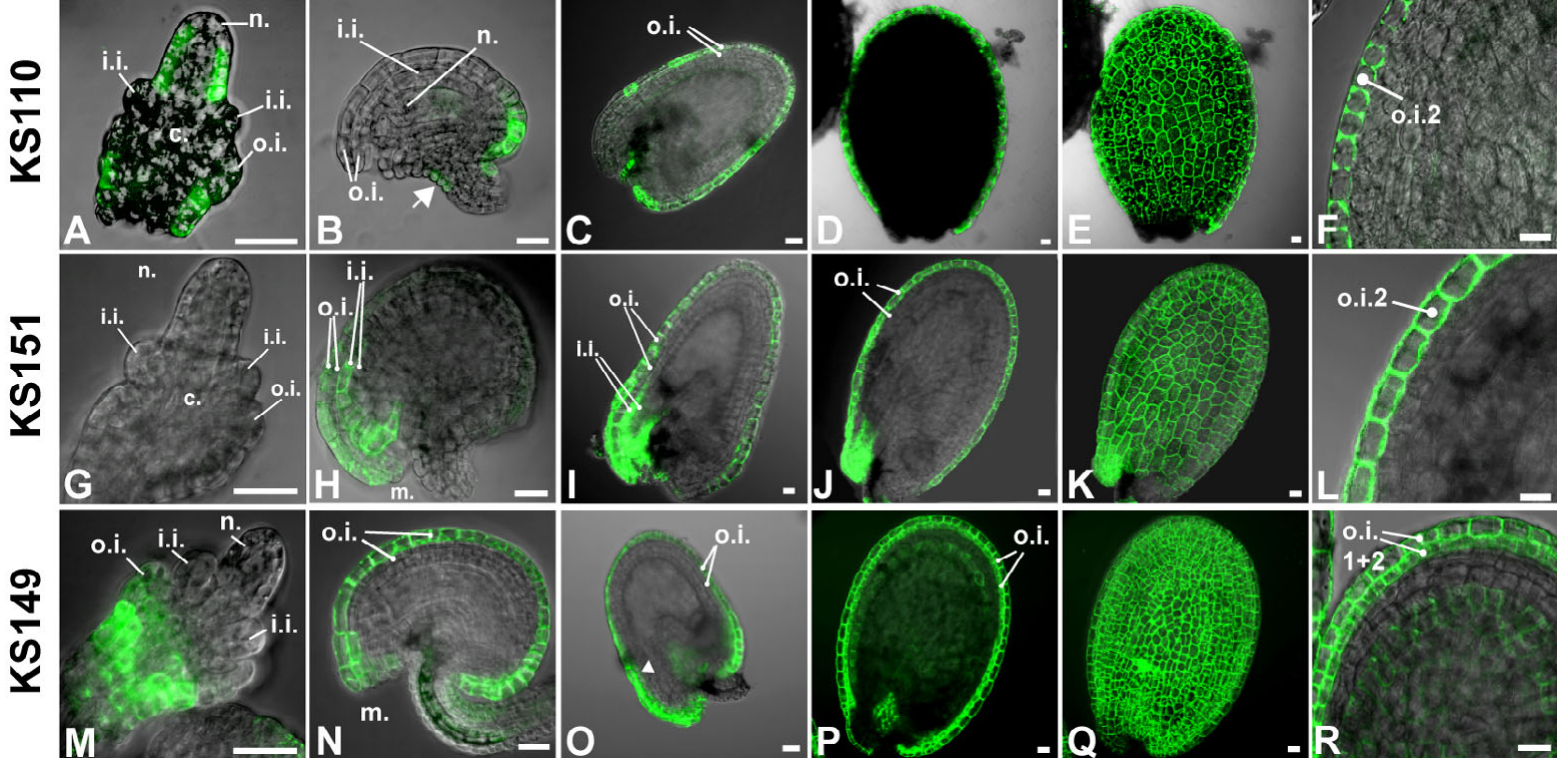
**Confocal laser-scanning images of *GFP *expression patterns during ovule and seed development in enhancer-trap lines KS110, KS151, and KS149**. **(A) **to **(F) ***GFP *expression in line KS110. **(A)**, **(B) **Ovule development: *GFP *is expressed in the abaxial layer of the outer ovule integument and in a subset of cells on the gynoapical side of the funiculus region (arrow). **(C) **to **(F) **Seed development: *GFP *is expressed throughout the o.i.2. **(G) **to **(L) ***GFP *expression in line KS151. **(G) **No *GFP *expression is seen during early ovule development. **(H) **After fertilization, GFP can be seen at the micropylar end of both integuments in the abaxial cell layers. **(I) **to **(L) **Late seed development: *GFP *is expressed on the micropylar end of the i.i.2 and throughout the o.i.2. **(M) **to **(R) ***GFP *expression in line KS149. **(M)**, **(N) ***GFP *is initially expressed only in the o.i.2. **(O) **to **(R) **During seed development, *GFP *expression is also seen in the o.i.1. The arrow in **(O) **shows beginning of expression in the o.i.1. **(E)**, **(K)**, and **(Q) **are overlay projection images of **(D)**, **(J)**, and **(P)**, respectively. **(F)**, **(L)**, and **(R) **show details of outer integument expression Scalebars: 20 μm.

*GFP *expression in ovules of line KS110 was restricted to the abaxial (outer) layer of the outer integument (o.i.2). Expression started before integument outgrowth in the epidermis of the funiculus (Figure 2A). As the outer integument grew out, *GFP *was first only expressed at the chalazal end (Figure 2B). During early embryogenesis, *GFP *expression extended throughout the o.i.2. Expression persisted in this layer during the later stages of seed coat development (Figure 2C to 2F). GFP was also found in the L1 layer of the nucellus during early stages of ovule development (Figure 2A). The expression in the abaxial layer of the outer integument resembled the expression of *INO *[16,17]. Unlike *INO *expression, however, the KS110 marker was also expressed in a small subset of cells in the epidermal layer on the gynoapical side (arrow in Figure 2B).

Line KS151 also showed *GFP *expression in the o.i.2. Expression started around the time of fertilization. In contrast to line KS110, *GFP *was initially expressed only at the micropylar end (Figure 2H). Later it could be seen throughout the outer layer of the outer integument (Figure 2I to 2L). In addition, line KS151 also exhibited *GFP *expression in the abaxial cell layer of the inner integuments, where expression remained restricted to the micropylar end throughout seed development.

In contrast to lines KS110 and KS151, line KS149 showed expression of *GFP *in both outer integument layers. GFP fluorescence was observed before integument outgrowth in the region immediately underneath the chalaza (Figure 2M). In the early stages of ovule development, *GFP *expression was only seen in the o.i.2 (Figure 2N). During early seed development GFP fluorescence then was also detected in the o.i.1. Expression of *GFP *remained in both outer integument layers during the late stages of seed coat development. Faint *GFP *expression was also seen in the endothelium cell layer (Figure 2P, R).

### The markers also label adaxial-abaxial cell layers in shoot tissues

**Figure 3:**
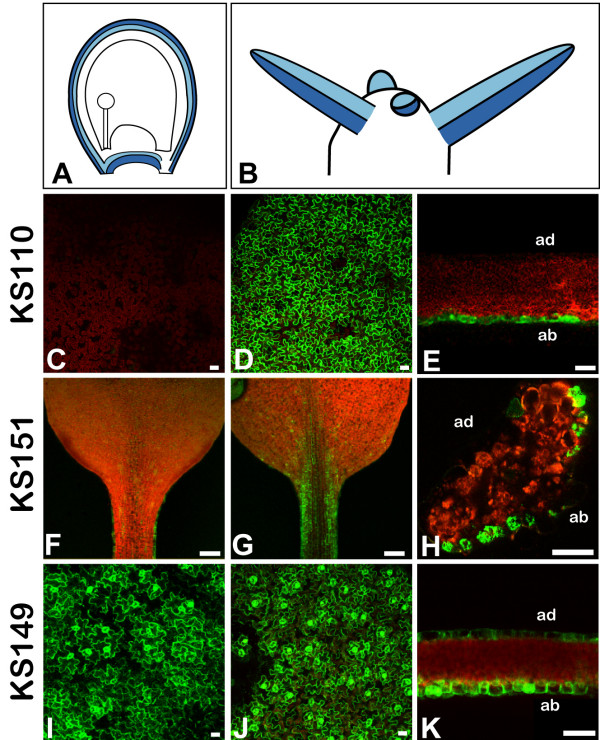
**Confocal laser-scanning images of *GFP *expression patterns in leaves of enhancer-trap lines KS110, KS151, and KS149 show similarities to ovule expression**. **(A) **Abaxial (dark blue) and adaxial (light blue) domains of the outer integument. **(B) **Abaxial and adaxial domains of lateral organs of the shoot apical meristem (colour code as in **(A))**. **(C) **to **(E) ***GFP *is only expressed in the abaxial domain of lateral organs in line KS110. **(C) **No *GFP *expression is seen in the adaxial epidermis of KS110 leaves (red colour is chlorophyll auto-fluorescence). **(D)**, **(E) **Strong *GFP *expression is seen in the abaxial leaf epidermis. **(F) **to **(H) ***GFP *is only expressed in the abaxial domain of lateral organs in line KS151. **(F) **Adaxial epidermis of leaf petiole showing no *GFP *expression. **(G)**, **(H) **Abaxial epidermis of petiole with *GFP *expression. **(I) **to **(K) ***GFP *expression in line KS149 marks abaxial and adaxial domains of lateral organs. Adaxial **(I) **and abaxial **(J) **leaf epidermis shows *GFP *expression. **(C)**, **(D)**, **(F)**, **(G)**, **(I)**, and **(J) **show surface views, while **(E)**, **(H)**, and **(K) **show sections through **(E)**, **(K) **the leaf lamina or **(H) **the petiole of the marker lines. Scalebars: 20 μm, in **(F) **and **(G)**: 100 μm.

Lines KS110, KS151, and KS149 showed expression of *GFP *in other lateral organs with the same axial preference as in the outer integument. In leaves and petals of KS110 plants expression of *GFP *was restricted to the abaxial epidermis (Figure 3C to 3E). Line KS151 showed *GFP *expression mainly in the leaf petiole. Again, expression was only found in the abaxial epidermis (Figure 3F to 3H) with erratic individual cells expressing *GFP *on the adaxial side. In KS149 leaves and petals, *GFP *was expressed strongly in the epidermis. Like in the outer ovule integument, it did not show any axial preferences (Figure 3I to 3K).

### A marker for the distal region of the abaxial outer integument cell layer

**Figure 4:**
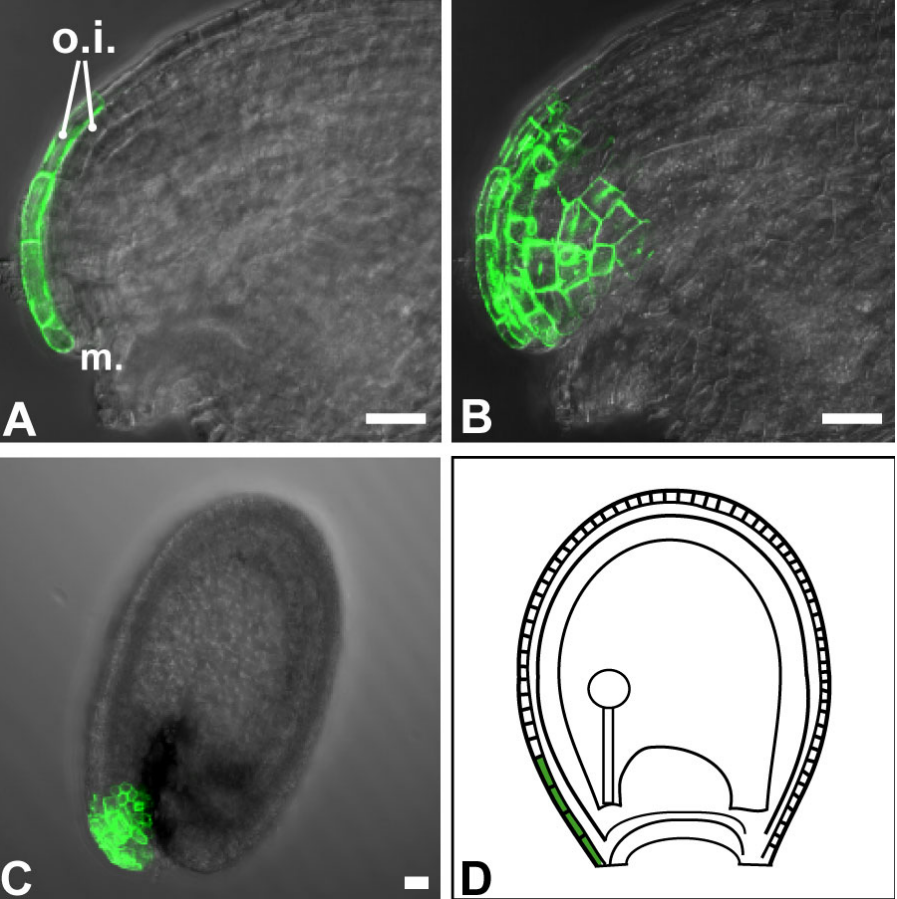
***GFP *expression in line M0237 during seed development**. *GFP *expression marks the long cells of the o.i.2 at the micropylar end of the outer integument. **(B) **Overlay projection of **(A)**. **(C) **Overlay projection of mature seed showing persistence of marker gene expression. **(D) **Cells with distinct identity are located at the distal end of the ovule and seed integuments. Scalebars: 20 μm.

Cells at the distal portion of the outer integument (the micropylar end) are visibly more elongated and are therefore distinct from the cells of the rest of the integument. GFP fluorescence in line M0237 was first detected around fertilization and was restricted to these cells throughout seed development (Figure 4). GFP expression in line M0237 therefore specifically marked this cell type. The M0237 marker was not expressed in leaves or petals.

### Cells in the adaxial and abaxial cell layer of the outer integument differ in size and shape

**Figure 5:**
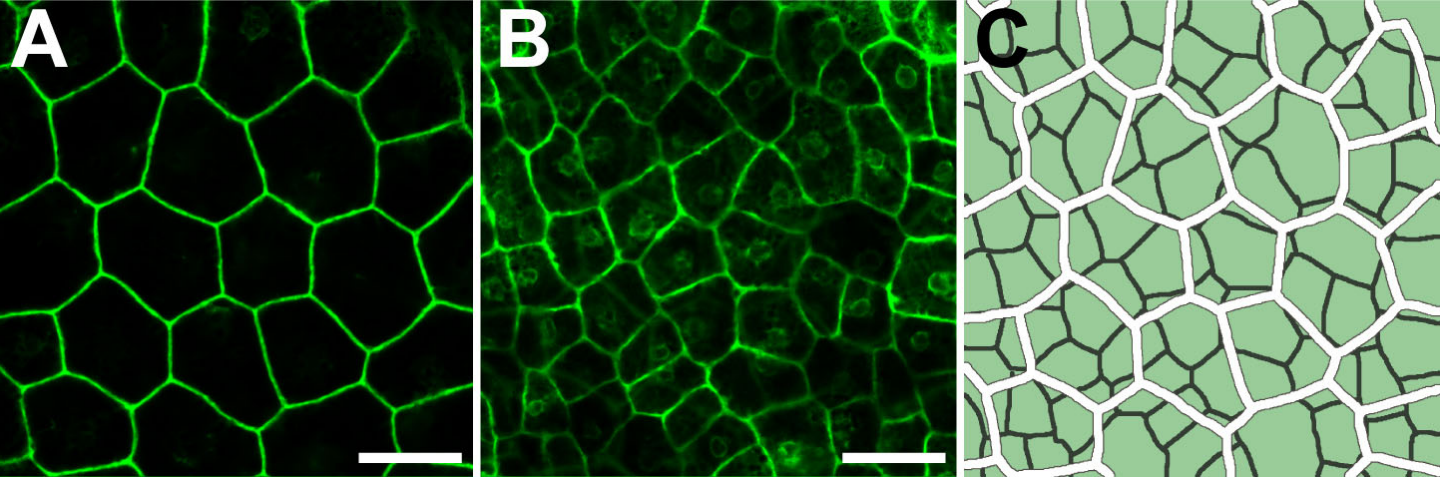
**Confocal laser-scanning images of *GFP *expression in the outer integument cell layers of line KS149**. *GFP *expression in the o.i.2 **(A) **and the o.i.1 **(B) **of line KS149. The embryo was at globular stage in this seed. **(C) **Overlay image of **(A) **and **(B)**, cell walls of the o.i.2 are coloured in white, cell walls of the o.i.1 in dark grey. Scalebars: 20 μm.

We took advantage of confocal microscopy, which makes it possible to image individual cell layers without the need for physical tissue sectioning. Line KS149, which shows GFP expression in both outer integument cell layers, was used to visualize cells in the o.i.1 and o.i.2. Seeds with globular stage (4 to 8 cell stage) embryos were analysed. Images of *GFP *expressing cells in the o.i.1 and o.i.2 were taken separately (Figure 5A, B). In 3 seed coats analysed, cell areas of the o.i.2 were significantly (p ≤ 0.0001) larger than those of the o.i.1 [see Additional file 1]. In addition, the majority of cells in the outer layer were 7-sided, while the inner layer had more 6-sided cells [see Additional file 1].

### Ectopic expression of *KNAT1 *causes extra cell divisions and reveals a compensatory mechanism during outer ovule integument morphogenesis

Over-expression of KNOX homeodomain proteins confers indeterminancy on normally determinate organs, such as leaves [12,13,18]. Ectopic expression of *KNAT2 *in ovules led to the homeotic conversion of the nucellus into carpeloid structures in a *Landsberg erecta *(*Ler*) background (Pautot *et al*. 2001). To investigate ovule development in *KNAT1 *over-expressing plants, the *KNAT1 *cDNA was translationally fused to the gene of the yellow fluorescent protein YFP and put under the control of the constitutive CAMV 35S promoter [19]. To ensure nuclear localization of KNAT1, a nuclear localisation sequence (NLS) derived from the SV40 T-antigen [20] was added to the *KNAT1*-*YFP *fusion. Twenty independent *Arabidopsis *lines (ecotype C24) were obtained. Eleven of the lines showed the characteristic lobed leaf phenotype that had been described previously for *KNAT1 *over-expressing plants [12,13]. Three lines with strong leaf lobing were chosen for further analysis of the T3 and T4 generation (lines 13, 41, and 51). Nuclear localized KNAT1-YFP fluorescence could be seen in all cells of the ovules of these lines throughout all development stages (not shown).

**Figure 6:**
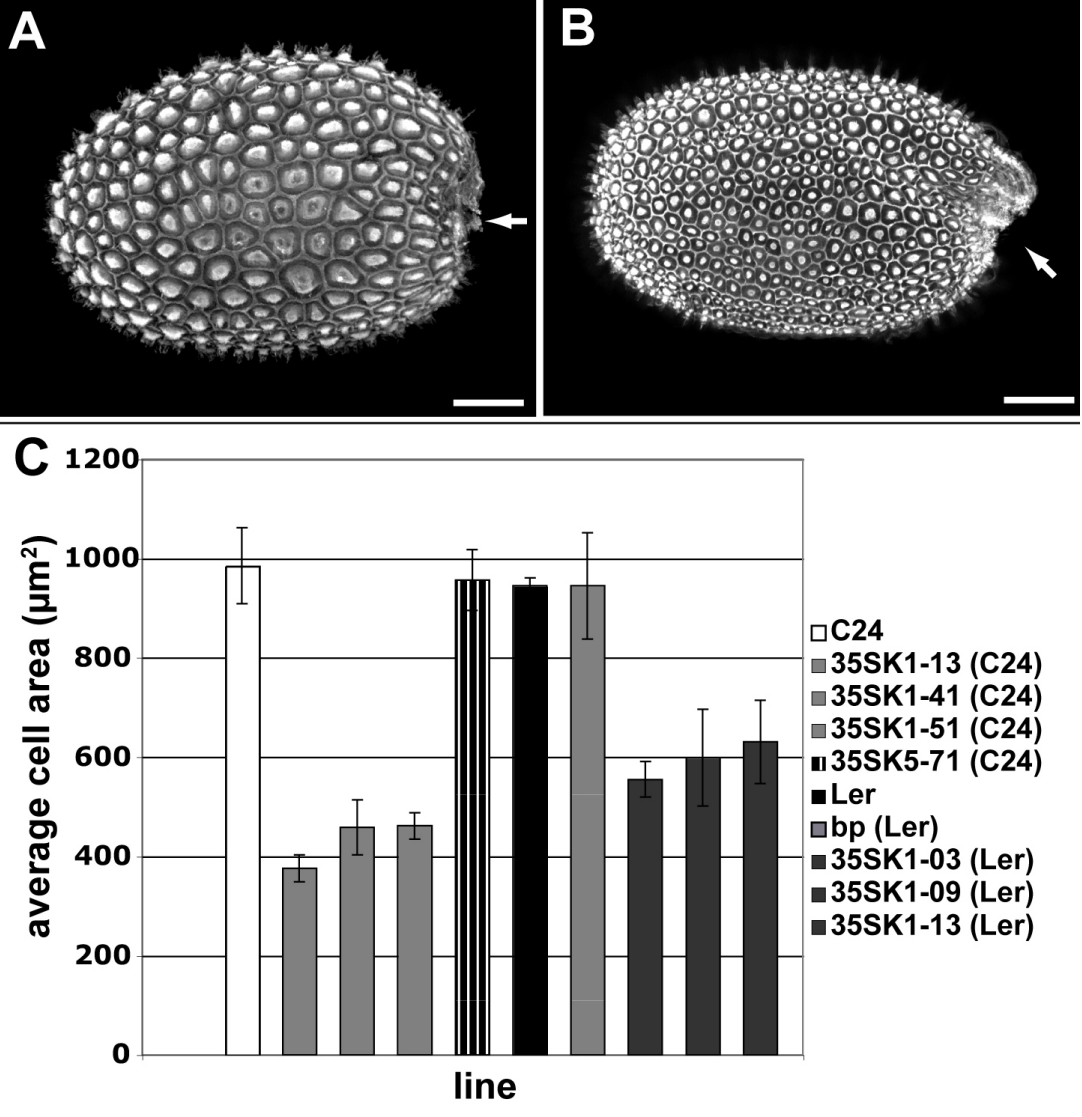
**Seed coat phenotype of *KNAT1 *misexpressing plants**. Confocal images of seed coats stained with safranin O. **(A) **Wild type seed coat. **(B) **Example of seed coats of a *KNAT1 *over-expressing line. Images are overlay projections. Arrows show the positioning of the integument closure. Scalebars: 100 μm. **(C) **Average area of cells in the outer seed coat of mature *Arabidopsis *seed of different transgenic lines and different ecotypes. Shown are data for ecotypes C24 and *Landsberg erecta *(*Ler*), for different KNAT1 overexpression lines (35SK1-xx) in C24 and *Ler *backgrounds, for a KNAT5 overexpression line (35SK5-71) in C24 background, and for *brevipedicellus *(bp) in *Ler *background. For each data point 25 – 30 cells of 3 different seeds were measured. Error bars show standard error of the means.

To analyse seed morphology, seeds were stained with the fluorescent dye safranin O and viewed with the confocal microscope. Seeds of the *KNAT1 *over-expressing plants showed two obvious morphological differences to wild type C24 seed:

1) The shape of an *Arabidopsis *wild type seed resembles an ellipsoid with the poles being at and opposite the side where the funiculus was attached. In wild type seed, the integuments closed up with the funiculus approximately in the middle of the funicular side of the seed. In seeds of *KNAT1 *over-expressers this closing was shifted towards the gynobasal side (see arrows in Figure 6A, B).

2) Seed coat cell sizes were obviously reduced in *KNAT1 *over-expressing lines (Figure 6A, B). Seed coat cell area sizes (n ≥ 25) of three seeds of three *KNAT1 *over-expressing lines were measured and compared to wild type. Cell areas in the seed coat of the *KNAT1 *over-expressing lines were about half the size of the wild type cell areas (Figure 6C). This difference was highly significant (p-values: 35SK1-13: 0.0025, 35SK1-41: 0.0052, 35SK1-51: 0.0036). Since seed sizes of wild type and *KNAT1 *misexpressing lines were not different (not shown), ectopic expression of *KNAT1 *thus caused the formation of about twice as many cells in the outer seed coat layer.

Reduced cell size was not a general feature of *KNAT1 *over-expression. Cell areas were measured in the abaxial and adaxial layers of the epidermis of mature petals (n of cells ≥ 22 per petal, 6 petals of 3 plants were analysed). No difference in petal cell area sizes could be detected between *KNAT1 *over-expressers and wild type (not shown).

To compare the *KNAT1 *overexpression phenotype with the phenotype reported for overexpression of *KNAT2*, we also introduced the *35S:KNAT1-YFP-NLS *construct into a *Ler *background. Reduced seed coat cell sizes similar to those observed in the C24 background were detected (Figure 6C), but no homeotic conversions were observed.

The seed coat was normal in plants transgenic for a *35S:KNAT5-YFP-NLS *construct (C24 background) (Figure 6C; [21]). Thus, we can exclude the formal possibility that YFP in the nucleus interferes with normal cell proliferation.

### Extra cell divisions in *KNAT1 *over-expressing plants occur specifically in the abaxial layer of the outer integument after fertilisation

To follow seed coat development in the *KNAT1 *over-expressing plants, we crossed the enhancer trap lines M0237, KS110, KS149, and KS151, and a line that constitutively expresses a plasma membrane localised form of *GFP *[22,23] into the *KNAT1 *over-expressing lines. For comparison, the marker lines were also backcrossed into C24. Developing ovules and seeds were analysed in the F1 generation.

**Figure 7:**
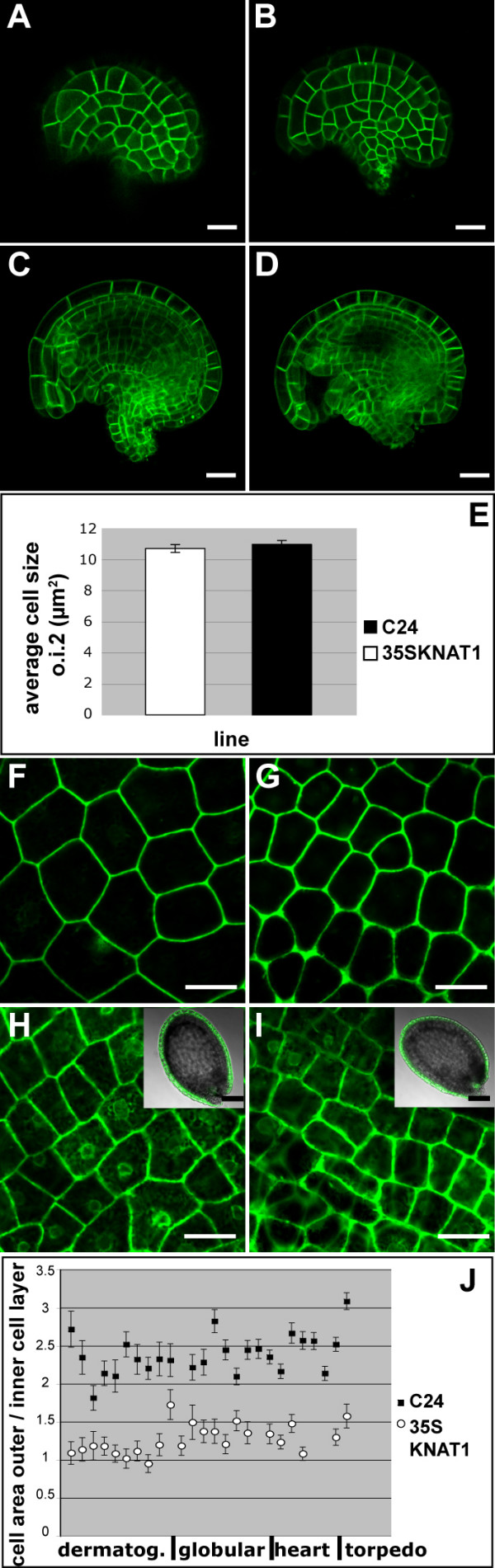
**Outer integument cell areas in wild type and *KNAT1 *misexpressing lines at different stages of development**. **(A) **to **(E) **Seed coat cell sizes of ovules at the stage of fertilization expressing a fusion between GFP and a membrane-localized protein. **(A) **Outer integument of wild type ovule. **(B) **Outer integument of ovule of *KNAT1 *misexpressing plant. **(C) **and **(D) **are optical sections of **(A) **and **(B)**, respectively. **(E) **Cell area measurements of o.i.2 cells shows no difference in cell area between wild type (black) and *KNAT1 *misexpressing (white) plants at this stage. **(F) **to **(J) **Seed coat cell area sizes of ovules after fertilization. **(F) **to **(I) **Seeds with globular stage embryos. **(F) **o.i.2 and **(H) **o.i.1 of line KS149. **(G) **o.i.2 and **(I) **o.i.1 of *KNAT1 *over-expressing plants crossed to line KS149. Cells in the o.i.2 of *KNAT1 *misexpressing plants are visibly smaller. **(J) **Ratio of cell area sizes in the o.i.2 versus the o.i.1 of wild type and *KNAT1 *misexpressing lines at different stages of seed development. Shown are measurements of integument cell areas (n = 25 – 30) of individual developing seeds related to the developmental stage of the embryo. Black squares: wild type. White circles: *KNAT1 *misexpressing lines. Scalebars: 20 μm, in inserts 100 μm.

The progeny of crosses of the membrane-marker line to the *KNAT1 *over-expressing plants were used to analyze cell area sizes of the abaxial layer (o.i.2) before fertilization. Cells in the outer layer of the outer integument had the same size in wild type and *KNAT1 *over-expressing plants (Figure 7A to 7E).

Crosses to line KS149 were used to analyse cell sizes of both outer integument layers after ovule fertilization. Images of both cell layers of seeds of different developmental stages were taken. Cell area measurements showed that only the cells in the abaxial layer of the outer integument of *KNAT1 *over-expressing plants were smaller than in wild type. From early embryo development onwards, the ratio of abaxial:adaxial cell area sizes was 2.4(+/- 0.328):1 in a wild type background and 1.27(+/- 0.194):1 in a *KNAT1 *over-expressing background (Figure 7F to 7J) (p-values for 3 arbitrary chosen data points: ≤ 0.0001). These numbers suggest that, on average, cells underwent one extra cell division in the o.i.2 of the *KNAT1 *over-expressing plants around or shortly after fertilization. Therefore, in *KNAT1 *over-expressing lines, the size of cells in the abaxial layer of the outer integument was more similar to those of the adaxial cell layer.

We can exclude that the different response to *KNAT1 *misexpression in the two cell layers of the outer integument was the result of different activities of the 35S promoter used for *KNAT1 *misexpression in those layers: The 35S promoter showed uniform expression throughout ovule development [see Additional file 2].

### Ectopic expression of *KNAT1 *causes altered marker gene expression

**Figure 8:**
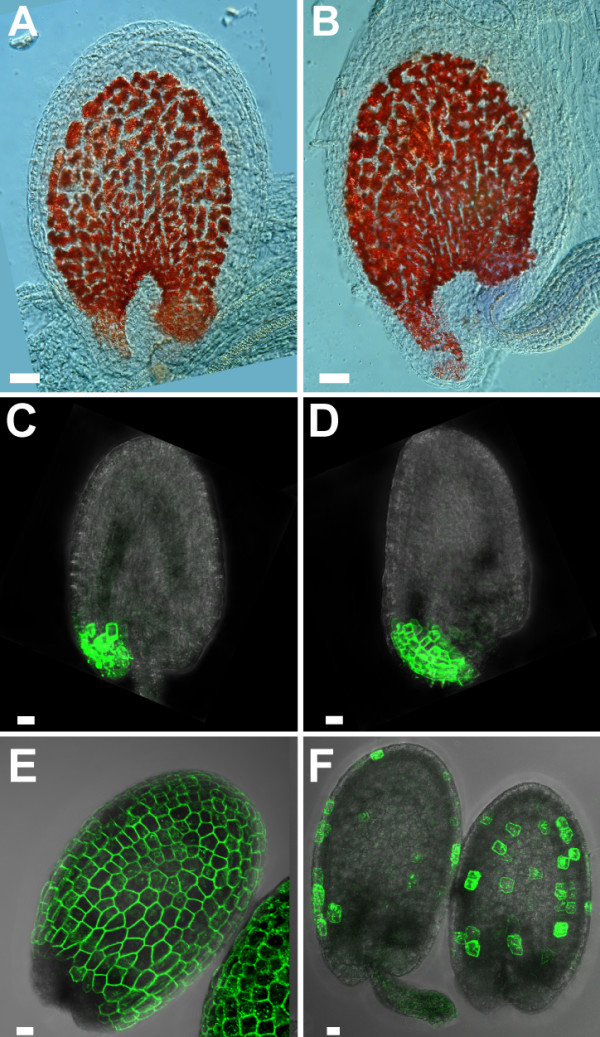
**Outer integument shape and cell fate changes in *KNAT1 *misexpressers**. **(A)**, **(C)**, **(E) **Wild type. **(B)**, **(D)**, **(F) ***KNAT1 *over-expressers. **(A)**, **(B) **Vanillin staining of the endothelium shows the altered shape of ovules of *KNAT1 *misexpressing plants. **(C)**, **(D) ***GFP *expression in crosses to line M0237. The domain of *GFP *expression in *KNAT1 *over-expressing plants is enlarged. **(E)**, **(F) ***GFP *expression in crosses to line KS110. Marker expression is repressed in plants misexpressing *KNAT1*. Scalebars: 10 μm

Crosses of line KS110 to the *KNAT1 *over-expressing lines resulted in plants with markedly reduced expression of *GFP *in the o.i.2. *GFP *was only expressed in a subset of random cells, and the outcome of the expression was later in relation to expression of *GFP *in a KS110 line that was backcrossed to C24 (Figure 8E, F). In contrast, the *KNAT1-YFP-NLS *fusion was normally expressed throughout the o.i.2, excluding cosuppression of the fluorescent protein genes (not shown). This shows an alteration of cell fate in the abaxial cell layer. Down-regulation of the KS110 marker was specific for the integument, as it was not observed in leaves of *KNAT1 *over-expressing plants.

### Over-proliferation of cells in the outer integument results in a more pronounced hyponastic growth of the integument

During wild type seed development, the micropylar end and the chalazal end of the integuments are approximately levelled. This is reflected by the shape of the endothelium. Figure 8A, B shows seeds of wild type and *KNAT1 *over-expressers during early embryo development. The endothelium accumulates proanthocyanidins and can therefore be selectively stained with vanillin. Unlike in wild type, the micropylar end was positioned below the chalazal end in the ovules of plants over-expressing *KNAT1*.

The same was seen in line M0237 that had been crossed to *KNAT1 *over-expressing plants. With ectopic expression of *KNAT1*, the region of *GFP *expression in M0237 at the micropylar end of the integument was shifted towards the gynobasal side. In addition, the *GFP *expression domain was longer than in wild type because more cells expressed *GFP *at the micropylar end (Figure 8C, D). Unlike the rest of the integument, cells at the micropylar end did not seem to be able to compensate fully for the extra cell division by reducing their size. Therefore more cells of almost normal size were expressing the *GFP *marker.

**Figure 9:**
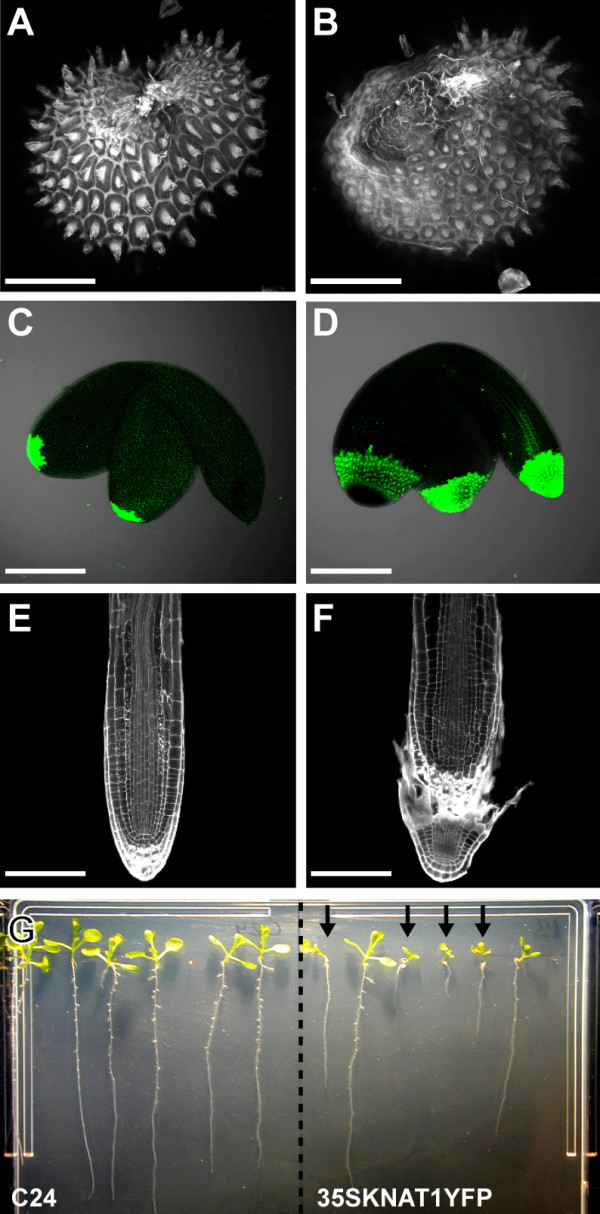
**Consequences of *KNAT1 *misexpression for seedling growth**. **(A)**, **(C)**, **(E) **Wild type. **(B)**, **(D)**, **(F) ***KNAT1 *over-expressing plants. **(A)**, **(B) **Closure of integuments in seeds stained with safranin O. The integuments are not fully closed in plants that misexpress *KNAT1*. **(C)**, **(D) **Sytox green staining of dead cells in embryos. Dead cells can be seen in the embryonic root tip of *KNAT1 *over-expressers. **(E)**, **(F) **3 day old roots stained with propidium iodide. Development of the embryonic root tip in *KNAT1 *over-expressers has not progressed. **(G) **Wild type and *KNAT1 *over-expressing plants grown on vertical plates for 10 days. Seeds were Na-hypochlorite treated for 10 min. Root growth of some *KNAT1 *over-expressing seedlings is impaired (arrows). Scalebars: 100 μm.

In summary, although reduced cell elongation largely compensated for the extra cell division in the o.i.2 of plants with ectopic expression of *KNAT1*, a more pronounced hyponastic growth of the integument was still noticeable. The shape of the seed of *KNAT1 *over-expressing plants was slightly distorted, with the closure of the integuments shifted towards the gynobasal side (see Figure 6A, B). As a result of the ovule bending, more tissue was exposed on the funicular end that was not protected by a seed coat (see Figure 9A, B).

In mature *Arabidopsis *seeds the embryo lies bent with the root tip at the micropylar end and the cotyledon tips at the chalazal end of the seed [24]. Therefore the root tip lies closest to the unprotected area of the *KNAT1 *over-expressing plant seed. We noticed that seedlings of *KNAT1 *over-expressing lines were often impaired in root growth and developed secondary roots at a much earlier stage (Figure 9G). These seedlings seemed to have localized tissue damage in the embryonic root tip and occasionally also at the tip of the cotyledons (Figure 9C to 9F). To quantify our observations, seeds (n = 30) of C24 and *KNAT1 *over-expressing lines were germinated and the number of seedlings with impaired root growth was counted. While wild type roots grew normally, 3.5% (line 41) and 7.1% (line 13) of seedlings of the *KNAT1 *over-expressers showed impaired growth. Sodium hypochlorite treatment, as commonly used for the surface sterilization of seeds, dramatically increased the number of seedlings with severely impaired root growth. Treatment with Na-hypochlorite for 5 minutes led to 20% and 36% seedlings with damaged root tips, respectively (wild type control: 3.5%). An exposure for 30 minutes resulted in more than 90% of seedlings with non-viable root tips in both transgenic lines (wild type control: 6.6%). Therefore, the seed coats of *KNAT1 *over-expressing plants provided less effective protection against the bleach solution, and cells at the site of closure of the integuments were most vulnerable to damage.

## Discussion

### Domains of gene expression in the outer integument

We have selected four *Arabidopsis *enhancer-trap lines [14] for the study of gene expression domains in the outer ovule integument. These lines expressed *GFP *under the control of endogenous enhancers, depending on the insertion of the enhancer-trap construct into the plant genome. The lines obtained in this study can be used for the analysis of ovule development, as has been shown here for the analysis of *KNAT1 *misexpressing ovules.

Two main points can be made from the study of *GFP *expression in the enhancer-trap lines: 1) The different cell identities of cells in the o.i.1 and in the o.i.2 are laid down during early integument development and are maintained. Lines KS110 and KS149 showed *GFP *expression in the o.i.2 but not in the o.i.1 of the developing outer integument in ovules before fertilization. *INO *also shows a similar expression pattern [16,17]. The establishment of polarity in the integuments might be important for the outgrowth of the integuments, as has been shown for leaves (for a recent review see [25]). In contrast to *INO*, *GFP *expression in lines KS149 and KS110 did not show axial preferences with respect to the gynoecium's axis. Lines KS110 and KS151 showed *GFP *expression in the outer layer of the outer integument also throughout seed development. The differences between o.i.1 and o.i.2 become obvious during late seed coat development when only cells of the o.i.2 differentiate into mucilage containing seed coat cells [3,4].

2) Cells at the micropylar end of both seed integuments are distinct from the rest of the integument cells. These cells also show obvious morphological differences, as they are noticeably longer than the average integument cells [1]. Moreover, they responded differently to *KNAT1 *misexpression. The morphological differences were reflected by the expression of markers that were not present in the rest of the integument. Line M0237 specifically marked cells at the micropylar end of the o.i.2 after fertilization and during seed development. Line KS151 marked this area in the outer cell layer of both integuments. It is likely that cells at the micropylar end have specific functions such as the protection of the embryo, which is growing from the micropylar end. Moreover, the region includes cells that are among the earliest to undergo elongation during integument growth. The differential expansion of these cells may help bend this tissue to form the characteristic shape of the ovule.

### Similarities of leaf and outer integument polarity

The expression patterns of our marker lines support the theory that the outer integument developed from a leaf-like structure [7,8]. While lines KS110, KS149, and KS151 with *GFP *expression in the outer integument did not show expression in the inner integuments, they all showed *GFP *expression in leaves and some also in other leaf-like structures, such as petals. Moreover, the markers that were expressed on the abaxial side of the outer integument (KS110 and KS151) also showed the same polar expression in leaves. So far, no gene has been found that is exclusively expressed in the o.i.1. To date, we also have not identified an enhancer-trap line that shows *GFP *expression solely in the o.i.1 (unpublished results). Surprisingly, it has been shown recently that *PHABULOSA *(*PHB*), a homeodomain gene that is expressed on the adaxial side of leaves [26,27], is expressed in the inner integument [28]. This led to the speculation that the two integuments of the bitegmic *Arabidopsis *ovule might have been derived through the splitting of one integument of a unitegmic precursor [28]. According to this, however, we would expect *GFP *expression in line KS149 to be found in both integuments, as *GFP *was expressed in all leaf cell layers in this line. In order to clarify these contradicting findings we need to investigate the expression patterns of more ovule-expressed genes on a whole plant level.

### KNAT1 overexpression phenotype

Many genes that play a role in SAM development are also important for ovule development and *vice versa*. Although *KNAT1 *is not normally expressed in ovules [13,29,30], it is likely that *KNAT1*, when expressed in the ovule, can interact with other proteins that are normal KNAT1 interaction partners in the SAM. Moreover, other *KNOX *genes could be expressed in the ovule and be involved in the regulation of meristematic activity during integument development. In many cases members of a gene family have become functionally diversified by changes in their expression patterns but their activities are still interchangeable. Several members of the YABBY family, for example, can restore integument outgrowth in *ino-1 *mutants. However, only the ovule expressed *INO *is sensitive to SUPERMAN (SUP) regulation that limits *INO *expression to the gynobasal side of the ovule primordium [17,31]. Accordingly, KNAT1 could partially adopt the role of another KNOX homeodomain protein but would be insensitive to proper regulatory mechanisms.

In the *Arabidopsis *SAM, KNAT1, together with STM1, plays a role in the transition zone where it allows for the amplification of stem cell daughter cells by maintaining their meristematic identity before they are consumed by organ formation [32]. In agreement with its role in the SAM, *KNAT1 *over-expression phenotypes in leaves have been interpreted as being the result of a shift from determinate to indeterminate growth characteristics [12,13,33]. In addition, the *KNAT1 *loss-of-function mutant *brevipedicellus *(*bp*) displays defects in cell division in internodes and pedicels, demonstrating that KNAT1 is also required for the maintenance of an indeterminate state in these organs [29,30]. Cell differentiation, elongation and growth in the pedicels of *bp *mutants were more severely affected on the abaxial side than on the adaxial side, causing a change in pedicel growth angle.

Expression of *KNAT1 *in the ovule integuments triggered ectopic cell divisions, consistent with a role of KNAT1 in maintaining indeterminancy. At present we cannot explain why the presence of KNAT1 only has a visible effect in the o.i.2. However, the phenotype of *bp *pedicels also seems to suggest that the abaxial side of pedicels is more affected by the presence of KNAT1 than the adaxial side. It is also possible that expression of *KNAT1 *in the integuments caused a certain degree of adaxialization of the outer integument and therefore the o.i.2 would adopt o.i.1 features. Two observations support this interpretation. First, expression of *KNAT1 *in the integuments caused cells of the o.i.2 to divide approximately as often as cells of the o.i.1. Second, a marker that is normally expressed in the o.i.2 but not in the o.i.1 was virtually absent in *KNAT1 *expressing ovules. As no markers are available that are only expressed in the o.i.1 we were unable to test the reverse situation.

### Regulation of cell proliferation during morphogenesis of the outer integument

We found clear differences in cell sizes in the two cell layers of the outer integument. Cell area sizes in the o.i.2 were 2.4 times bigger than in the o.i.1, which indicates that during integument development cells in the adaxial layer undergo approximately one division more than cells in the abaxial layer.

Misexpression of *KNAT1 *almost abolished the differences of cell area sizes in the two layers. Formally, the higher number of smaller cells found in the o.i.2 of integuments of *KNAT1 *over-expressing plants could be the result of impaired cell elongation or increased cell division. It is more likely that KNAT1 promoted cell division, since cells of the o.i.2 were still able to elongate after the extra cell division had already occurred. A compensatory mechanism was in place in the o.i.2 that resulted in the restriction of cell expansion and therefore in the development of an outer integument of relatively normal size and shape.

During leaf morphogenesis, mechanisms exist to compensate for defects in cell division or expansion (for review see [34,35]). As a general rule it can be stated that a decrease in cell number or an increase in cell elongation can be compensated for by increased cell expansion or decreased cell division, respectively. So far, there are only a few examples that an increased number of cell divisions or decreased cell expansion, as seen here for *KNAT1 *expression in the o.i.2, could lead to similar compensatory mechanisms [35,36]. To our knowledge a cell layer specific increase of cell divisions, which would more accurately correspond to the *KNAT1 *overexpression phenotype, has not been achieved so far. Therefore, the *KNAT1 *overexpression phenotype of ovules represents the first description of a compensatory mechanism that involves a decrease in cell elongation to compensate for increased cell divisions in one specific cell layer. Strict control of integument size on the whole organ level and cell-cell communication between the o.i.1 and the o.i.2 has to be postulated.

For *Arabidopsis *it has been shown that endosperm growth has a reciprocal effect on integument cell elongation to control final *Arabidopsis *seed size [37]. Here we show that, in addition to that, growth in the two outer integument cell layers is precisely and independently regulated to achieve coordinated integument morphogenesis. The outer integument would therefore be a good system to study the mechanisms of coordinated cell proliferation that result in the final shape of a plant organ.

## Conclusion

This work shows that previously not recognized compensatory mechanisms exist to ensure proper ovule integument morphogenesis. Moreover, we demonstrate that the outer ovule integument can be used as a good model system to study the basic principles of plant organ morphogenesis. The outer integument is a simple two-cell layered structure, consists of only a few different cell types, and is easily accessible by confocal microscopy. A marker line expressing *GFP *in both layers of the outer integument enabled us to measure cell areas in the two outer integument cell layers of developing *Arabidopsis *wild type seeds and can be used for a high-throughput screen for mutants with altered cell division patterns in the integument. Misexpression of *KNAT1 *in the ovule produces a clear and easily visible phenotype that can be used to identify factors involved in the regulation of cell division and elongation on the organ level.

## Methods

### Transgenic lines

The GAL4-GFP enhancer trap lines [14] KS110, KS149, KS151, and M0237 are available from the Nottingham *Arabidopsis *Stock Centre (NASC) [38] as stock numbers N9260, N9266, N9267, and N9339, respectively. Other transgenic lines obtained from the NASC: *bp-1 *(NW30).

### Construction of transgenic plants

The generation of the *KNAT1 *over-expression construct has been described [21]. Plasmids were electroporated into *Agrobacterium tumefaciens *GV3101 [39]. *Arabidopsis thaliana *ecotype C24 and Ler was transformed by floral dip [40]. Transgenic plants were selected on media containing 50 mg/l kanamycin.

### Growth conditions

Plants were germinated and grown under a 16 h light, 8 h dark photoperiod on media containing 0.5× Murashige and Skoog salt mixture (MS), 0.5 g/l 2-(N-morpholino) ethanesulfonic acid (MES) pH 5.7 and 0.7% agar. For analysis of ovule development plants were grown on soil under constant conditions in the greenhouse.

### Confocal laser-scanning microscopy

Confocal laser-scanning microscopy was performed using a Leica TCS NT/SP microscope. Excitation wavelengths were 488 nm for GFP, and 514 nm for YFP.

### Seed coat staining

For staining of mature seed coats with safranin O, seeds were incubated in a 1:10^5 ^dilution of safranin O (Molecular Probes, Eugene, USA) for 15 min at room temperature. Seeds were imaged with the confocal laser-scanning microscope with an excitation wavelength of 488 nm and a collection window of 540 – 600 nm. Endothelium staining with vanillin was performed according to Nesi et al. [41].

### Cell area measurements

To measure seed coat cell areas, optical sections were taken with the confocal laser-scanning microscope through the outer ovule integument. Cell area measurements were performed on a Macintosh computer using the public domain NIH Image programme (developed at the U.S. National Institutes of Health and available on the Internet [42]).

To measure petal cell areas, petals were cleared with chloral hydrate and images were taken at the microscope with a digital camera. Petal cell areas were measured as described above.

### Sodium-hypochlorite treatment

For seed sterilization and as a functional test of the seed coat, seeds were imbibed in a solution of 2.4% active sodium hypochlorite (for incubation times see results section). Seeds were washed 2 times with sterile water and plated on growth media.

## Authors' contributions

ET designed, coordinated and carried out the experiments and drafted the manuscript. JH participated in the coordination of the experiments and in drafting the manuscript. All authors read and approved the final manuscript.

## Supplementary Material

Additional file 1 **Size and shape of cells in the outer ovule integument**. **(A) **Cell area measurements of cells in the o.i.2 and o.i.1 of seeds with globular stage embryos show highly significant differences in cell sizes between the layers. **(B) **Distribution of cell shapes in the two outer integument cell layers. Click here for file

Additional file 2 **Activity of 35S promoter in the ovule is uniform**. Ovule development visualised in a line that expresses GFP in the plasma membrane under control of the constitutive 35S promoter. The 35S promoter shows uniform expression throughout the developmental stages. Scalebars: 20 μm. Click here for file
