# Supplementary figures and images for: Arabidopsis thaliana outer ovule integument morphogenesis: Ectopic expression of KNAT1 reveals a compensation mechanism
Source: BMC Plant Biol. 2008 Apr 14;8:35. doi: 10.1186/1471-2229-8-35 (PMC2330050; doi:10.1186/1471-2229-8-35)

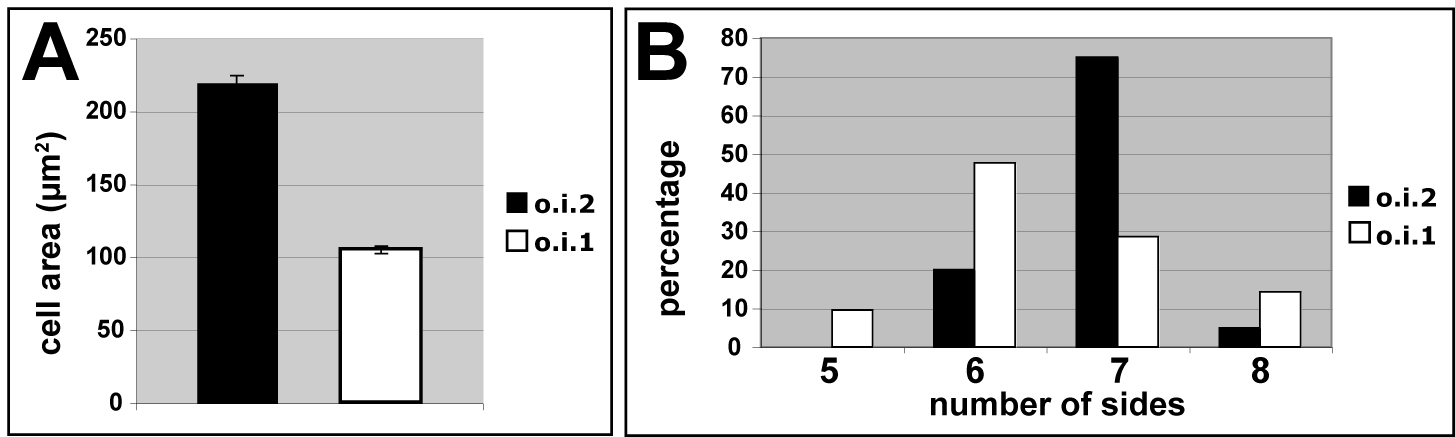

Supplement: Additional file 1 — Size and shape of cells in the outer ovule integument. (A) Cell area measurements of cells in the o.i.2 and o.i.1 of seeds with globular stage embryos show highly significant differences in cell sizes between the layers. (B) Distribution of cell shapes in the two outer integument cell layers. [file 1471-2229-8-35-S1.jpeg]

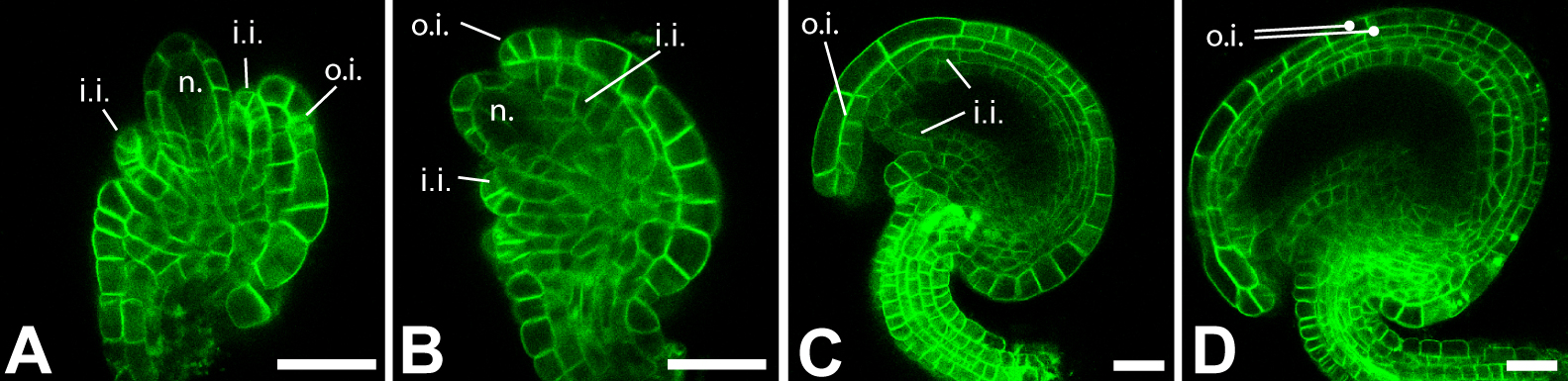

Supplement: Additional file 2 — Activity of 35S promoter in the ovule is uniform. Ovule development visualised in a line that expresses GFP in the plasma membrane under control of the constitutive 35S promoter. The 35S promoter shows uniform expression throughout the developmental stages. Scalebars: 20 μm. [file 1471-2229-8-35-S2.jpeg]
